# Monthly Summary

**Published:** 1884-06

**Authors:** 


### Monthly Summary.

Dental Irritation Causing Spasms.—At the meeting on March 20th, of the Harveian Society of London, Mr. Henry Sewill read a paper on Spasms of Muscles of the Face and Cataract due to Dental Irritation. The subject was a middle-aged lady, laboring under inveterate neuralgia, who during late years had suffered from a series of alveolar abscesses, and in 1881 had complained of recurring pains in the right eye, complicated since January, 1882, with persistent spasm of the right side of the face.

In July, 1882, partial loss of sight from right cataract, and partial closure of the same eye from spasm of the orbicularis, were superadded.

In July, 1883, at the request of her medical adviser, Dr. W. Hoffmeister, she consulted Dr. Ferrier, who, suspecting the symptoms to be due to dental disease, prevailed upon her to submit to the extraction of the offending teeth. Mr. Sewill found in the upper jaw the stumps of some molars, and artificial bicuspids, incisors and canines attached to a vulcanite plate, which had not been removed for years. The right canine was completely broken down, and pus exuded from several fistulous openings in the gums. The lower teeth were hidden beneath an enormous mass of tartar.

The treatment consisted in the extraction of all the upper teeth at two sittings, and in gradual removal of the tartar from the lower teeth. Within two days of the extraction the facial spasm entirely disappeared, but no change took place in the damaged lens.

Mr. H. Power had lately stated in a paper read before the Odontological Society that dental lesions are very frequently the cause of diseases of the eye.

Threatening glaucoma, especially when associated with ciliary neurosis, and with obscure temporal and orbital pain, mydriasis, myosis, sudden paralysis of some of the orbita, muscles, and loss of sensation in the absence of cerebral symptoms, phlyctenulæ and corneal ulcers (especially in children), sudden failure of accommodation and exophthalmos, were conditions calling for careful inspection of the teeth. To these should now be added cataract.

Mr. Power had pointed out various ways in which irritation originating in the teeth might reach the eye: (1) Inhibition; (3) trophic or vaso-motor influence; (3) extension of neuretis; (4) reflex irritation or sympathy.

The case recorded above was probably an instance of trophic disturbance.

The President called attention to the frequency with which the ear was affected from the same cause; the subject was fully discussed in a recent work by Dr, Samuel Sexton, of New York.

Mr. F. Treves referred to cases similar to that brought before the Society, which had occurred in the practice of Mr. Hilton and Dr. G. Johnson. Addressing himself to the anatomical aspect of the question, he criticised the theories suggested by the author for an explanation of the nervous mechanism at work. The case might lend support to the view that a nervous communication existed between the second division of the fifth nerve and the lenticular gangloin.

Mr. Juler, from observations upon a large number of cases in which eye affections were associated with disease of teeth, was of opinion that the latter were even more commonly the cause of ocular and intra-ocular lesions than Mr. Sewill appeared to consider.

As illustrative cases he would mention that of a young lady, aged seventeen, who was sent to him by Dr, Jennings, of Notting-hill. See was otherwise in good health; the right eye was slightly hypermetropic, but the vision bad, and the ophthalmoscope showed patches of recent choroiditis at the posterior pole of this eye.

No cause could be found for the lesion until her dentist, Mr. Percy May, informed him that she was suffering from a carious tooth in the left upper jaw. The tooth was extracted, with considerable improvement in the vision.

Another case had occurred lately in a young lady who was sent to him from Bedford. In this case the vision of the left eye was reduced to the perception of fingers only; she had evident symptoms of inflammation of the ciliary region, and the vitreous was crowded with large membranous and fine floating opacities.

No cause could be found for the ocular trouble until a history of neuralgia led to the discovery of carious teeth. She had promised to have the teeth extracted, and Mr. Juler had no doubt that the eye would afterwards improve.

A delicate woman, aged fifty, consulted him at the Westminister Ophthalmic Hospital a year ago on account of persistent jactitation of the right eyelids. The case was an interesting one, inasmuch as there was a "point of election" just opposite the infra-orbital foramen, over which firm digital pressure had the effect of immediately stopping the movements. A carious tooth in the upper jaw of the same side was extracted, and with it disappeared the facial trouble.—*London Lancet.*

---

A NEW REMEDY FOR SPASMS.—The *Med. Central. Zeit.*, of July 15th, 1882, publishes the result of experiments instituted by Dr. Schifier, of Berlin, for the purpose of determing the therapeutic value of the *extract of Guachamaca*, a tree indigenous on the Apuche mountains. As a specific for all spastic conditions of the motar apparatus we so far possessed, in reality, one remedy only; curace, which, however, on account of its danger, and the uncertainty of its action, as well as of the remedy itself, has mostly been used for physiological experiments alone, and, perhaps, occasionally in tenanus.

Guachamaca extract, prepared from the bark of the Quebracho plant, which belongs to the same class as the Oleander (Nerium ol. L.) and of which latter the skin between the tree and bark is also poisonous, is a remedy which, while not so dangerous in its effects, is far more reliable and uniform in its action, and can beside easily be procured pure and genuine, and of always equal strength, as it is by no means rare.

Schiffer experimented with this drug, and made his observations in the clinic of Prof. Frerichs. He employed the remedy in solution, in the dose of ⅙ of a grain, and by the hypodermic method, in cases of tonic and clonic spasms of the muscular system, and always with a very good effect. He noted also that, administered internally, no matter in what solution, the drug was as little absorbed by the mucous membrane of the alimentary canal as curare is.

Should further trials with this remedy prove invariable as successful, or at least frequently so, as was the case in Schiffer's experiments, we would at last possess a reliable specific for all spasmodic affections of muscles and motar nerves, a drug, the physiological action of which would be exactly opposite to that of strychnine and similar remedies.—*Med. and Sur. Reporter.*

---

ETHER.—In the *Lancet*, Dr. H. Bendelack Hewetson discusses the relative value of ether when prepared with "rectified" or "methylated" spirits of wine. The author states that there is a great difference between them. That prepared with rectified spirits has been found by those who have used it less safe and less desirable, producing more sickness and laryngeal spasm in certain cases in which there is a tendency to such complications. Of the use and applicability of the methylated ether—as the safest anæsthetic known, where carefully administered by means of Clover's inhaler—he can speak strongly, as the result of daily observation. It is a very ordinary circumstance to accupy eight seconds in producing complete anæsthesia, without a struggle or a cough, and it is by no means extraordinary for a patient to be "fully under" *within* the minute. In the case of short operations upon the eyes, and the like, it is hardly ever necessary to reapply the inhaler after it has been once removed for the operator to commence, the patient remaining sufficiently anæsthetic for an operation such as mentioned to be completed without hurry. Anæsthesia can be prolonged with equal safety even so far as to keep a patient in labor completely under its influence for upward of *four hours*, the longest time which has happened in my experience. Methylated ether is, I consider, from this point of view, the safest and cheapest anæsthetic at present in use.—*Med. and Surg. Reporter.*

EFFECT OF TOBACCO ON BOYS.—Dr. Edward C. Otis says: that the evil effects of tobacco upon boys which can be said to have been fairly proved by observation, are:

1. An interference with and impairment of the general development, mentally and physically. Probably it does this by retarding progressive cell change and impairing nutrition. In an adult this development has been attained; consequently he can smoke with impunity so far as this effect is concerned, when the boy only does it with injury.

2. The tobacco heart. This likewise happens to the adult; but is, I should think, more likely to happen to the youth, and with graver result, because his is such an unstable age, physically. For this same reason the other injurious effects are more liable to be intensified in the youth.

3. Defective muscular co-ordination. I have but little proof to show that this condition is more than temporary, continuing only while the tobacco is used.

4. It reduces the intellectual power of the boy. It does this either by opposing mental application and effort, or else by producing deterioration of the intellect—probably both to a greater or less intellect.

5. It impairs the memory. What proof I have adduced seems to show that this is a permanent injury.

6. It may cause defective vision and also irritation, more or less chronic, to the external part of the eye.

7. It may unduly excite the sexual system and lead on to excesses. I have no positive proof of this, but my observation among boys rather points to it.

8. Irritation of the mucons membrane of the mouth and throat, more especially in cases of cigarette smoking.

9. It may impair digestion and produce other derangements, such as habitual uneasiness, hypochondriasis, etc. —*Boston Medical and Surgical Journal.*

---

EXCISION OF THE TONGUE.—Mr. Croly, of Dublin, performs the following operation for cancer of the tongue, even when the disease is situated in the anterior portion of the organ. He first ligatured each lingual artery close to the hyoid bone, through a curved incision, reaching from the symphysis down to the hyoid bone, and up and back to the angle of the jaw. Through these incisions he withdraws the tongue, as in Regnoli's operation, and removes the requisite amount of it by the benzoline cautery. Lastly, he divides the gustatory nerve where it lies along the inner border of the jaw-bone.—*Med. and Sur. Reporter.*

---

TRIAL PLATES FOR THE LOWER JAW.—These often give us a vast deal of vexation, because, if not made stiff, they bend and displace the teeth they carry, much to our annoyance and loss of time. To obviate all these troubles, Dr. Samuel Wardle recommends that the plate be made of modeling composition, (medium grade) and strengthened with twisted wire. Ordinary iron wire is annealed and two strands twisted together, the result being a wire that looks like a thin screw. This is cut into lengths of about three inches and kept on hand for ready use. Two pieces are generally required to stiffen a plate. Bend them to suit the form of the plate, warm in the spirit flame or gas jet, and press them into place in the composition. When cold, the plate is as stiff as thick lead; made in one-third the time and without injury to the model. Try it. —*Dental Register.*

